# Targeting the Clear Cell Sarcoma Oncogenic Driver Fusion Gene *EWSR1::ATF1* by HDAC Inhibition

**DOI:** 10.1158/2767-9764.CRC-22-0518

**Published:** 2023-07-03

**Authors:** Hirokazu Mae, Hidetatsu Outani, Yoshinori Imura, Ryota Chijimatsu, Akitomo Inoue, Yuki Kotani, Naohiro Yasuda, Sho Nakai, Takaaki Nakai, Satoshi Takenaka, Seiji Okada

**Affiliations:** 1 Department of Orthopedic Surgery, Osaka University Graduate School of Medicine, Osaka, Japan.; 2 Center for Comprehensive Genomic Medicine, Okayama University Hospital, Okayama, Japan.; 3 Department of Orthopedic Surgery, Osaka National Hospital, Osaka, Japan.; 4 Musculoskeletal Oncology Service, Osaka International Cancer Institute, Osaka, Japan.

## Abstract

Clear cell sarcoma (CCS), a rare but extremely aggressive malignancy with no effective therapy, is characterized by the expression of the oncogenic driver fusion gene *EWSR1::ATF1*. In this study, we performed a high-throughput drug screening, finding that the histone deacetylase inhibitor vorinostat exerted an antiproliferation effect with the reduced expression of *EWSR1::ATF1*. We expected the reduced expression of *EWSR1::ATF1* to be due to the alteration of chromatin accessibility; however, assay for transposase-accessible chromatin using sequencing and a cleavage under targets and release using nuclease assay revealed that chromatin structure was only slightly altered, despite histone deacetylation at the EWSR1::ATF1 promoter region. Alternatively, we found that vorinostat treatment reduced the level of BRD4, a member of the bromodomain and extraterminal motif protein family, at the EWSR1::ATF1 promoter region. Furthermore, the BRD4 inhibitor JQ1 downregulated EWSR1::ATF1 according to Western blotting and qPCR analyses. In addition, motif analysis revealed that vorinostat treatment suppressed the transcriptional factor SOX10, which directly regulates *EWSR1::ATF1* expression and is involved in CCS proliferation. Importantly, we demonstrate that a combination therapy of vorinostat and JQ1 synergistically enhances antiproliferation effect and *EWSR1::ATF1* suppression. These results highlight a novel fusion gene suppression mechanism achieved using epigenetic modification agents and provide a potential therapeutic target for fusion gene–related tumors.

**Significance:**

This study reveals the epigenetic and transcriptional suppression mechanism of the fusion oncogene *EWSR1::ATF1* in clear cell sarcoma by histone deacetylase inhibitor treatment as well as identifying SOX10 as a transcription factor that regulates *EWSR1::ATF1* expression.

## Introduction

Clear cell sarcoma (CCS) is an exceedingly rare subtype of soft-tissue sarcoma (STS) that usually occurs in the lower extremities of adolescents and young adults (1). Previously, CCS was considered a melanoma of soft parts but was later distinguished from malignant melanoma by the presence of a specific fusion oncogene, *EWSR1::ATF1*, which is derived from a chromosomal translocation, t(12;22)(q13;q12) (2, 3), and essential for both the development and maintenance of CCS (4, 5). The standard treatment for CCS involves surgical resection with an adequate margin. Nevertheless, around half of patients with CCS develop distant metastases, and the 5- and 10-year overall survival rates are reportedly 50% and 38%, respectively (6). Systemic therapies are adapted to such patients, but conventional chemotherapy and radiotherapy have limited beneficial effects on CCS (7, 8). Therefore, to improve clinical outcomes, novel effective antitumor drugs are urgently needed.

In this study, we conducted high-throughput screening for CCS treatment, finding that vorinostat, the first pan histone deacetylase inhibitor (HDACi) approved by the FDA for the treatment of cutaneous T-cell lymphoma in 2006, is a potentially effective therapeutic agent against CCS. Interestingly, we found that vorinostat suppresses the expression of *EWSR1::ATF1* in CCS cells. Indeed, previous studies revealed that other HDACis suppress the expression of tumor-specific fusion oncogenes in CCS and other STSs (9–11). However, the suppression mechanism remains unclear. Elucidating this mechanism might facilitate the development of fusion gene–targeted therapy, which is an ideal approach for treating translocation-related sarcomas. Therefore, we focused on elucidating the epigenetic and transcriptional mechanisms underlying fusion gene suppression in CCS. Furthermore, we identify another drug for enhancing *EWSR1::ATF1* suppression in CCS.

## Materials and Methods

### Cell Culture

The human cell lines Hewga-CCS (CCS; RRID:CVCL_0J32), Asra-EPS (epithelioid sarcoma; RRID:CVCL_W949), Yamato-SS (synovial sarcoma; RRID:CVCL_6C44), and Kitra-SRS (CIC-DUX4 sarcoma; RRID:CVCL_YI69) were established in our laboratory. MP-CCS-SY (CCS; RRID:CVCL_0J33) and KAS (CCS) were kindly provided by Dr. Moritake (Miyazaki University, Miyazaki, Japan) and Dr. Nakamura (Japanese Foundation for Cancer Research, Tokyo, Japan). SYO-1 (synovial sarcoma; RRID:CVCL_7146) and HS-SY-II (synovial sarcoma; RRID:CVCL_8719) were kindly provided by Dr. Ozaki (Okayama University, Okayama, Japan) and Dr. Toguchida (Kyoto University, Kyoto, Japan). NEPS (epithelioid sarcoma; RRID:CVCL_IS66) was kindly provided by Dr. Kabata (Kanazawa University, Ishikawa, Japan). SU-CCS1 (CCS; RRID:CVCL_B470), VAESBJ (epithelioid sarcoma; RRID:CVCL_1785), HT-1080 (fibrosarcoma; RRID:CVCL_0317), and SW872 (liposarcoma; RRID:CVCL_1730) were purchased from ATCC. Normal human dermal fibroblasts (NHDF) were purchased from Kurabo. All cell lines were cultured in DMEM (Nacalai Tesque) containing 10% FBS (Sigma–Aldrich) at 37°C with 5% CO_2_ under 100% humidity. All cell lines were authenticated by examination of morphology, genotyping by PCR and growth characteristics, and were used between passages 10 and 30. The cell lines were verified to be negative for *Mycoplasma* contamination using TaKaRa PCR Mycoplasma Detection Set prior to experiments.

### High-throughput Screening

The Hewga-CCS cell line was used for high-throughput screening. Cells were seeded at 20,000 cells/well in 384-well culture plates in DMEM containing 10% FBS and cultured overnight at 37°C in a humidified atmosphere with 5% CO_2_. The cells were then exposed to 1,134 FDA-approved drugs (10 μmol/L each) in our library (Selleck Chemicals) using a FLUENT High-Throughput Assay System (TECAN), and cell viability was measured 48 hours later using a Cell Counting Kit-8 (Dojindo).

### Compounds

Vorinostat was purchased from Santa Cruz Biotechnology (#sc-220139), romidepsin and mivebresib were purchased from Selleck Chemicals (#S3020, #S8400), and JQ1 was purchased from Chem Scene (31268524–70–4). The drugs were dissolved in DMSO (Sigma-Aldrich), after which they were added to cell cultures for further investigation according to the manufacturer's instructions.

### WST-8 Cell Proliferation Assay

CCS cell lines were seeded into 96-well plates at a density of 5 × 10^3^ in triplicate and incubated with agents or the vehicle for 48 hours. The cell proliferation rate was measured using Cell Count Reagent SF (Nacalai Tesque). Absorbances at 450 and 690 nm (reference wavelength) were measured using a microplate reader, and the relative cell proliferation rate was calculated.

### Flow Cytometry

CCS cell lines were seeded at a density of 1 × 10^6^ per 10-cm dish and cultured for 24 hours, after which vorinostat or vehicle were added as a treatment. After 48 hours of treatment at the indicated concentration, the cells were harvested and stained with a propidium iodide (PI) solution (25 μg/mL PI, 0.03% NP-40, 0.02 mg/mL RNase A, and 0.1% sodium citrate) for 30 minutes at room temperature. A BD FACSVerse flow cytometer (Becton Dickinson) and the BD FACSuite Software Application (Becton Dickinson) were used to analyze the cell cycle according to the manufacturer's protocol.

### Western Blot Analysis

CCS cells were seeded at a density of 4 × 10^5^ cells/well in 6-well plates and incubated with vorinostat or the vehicle at the indicated concentration for 24 hours. For lysate preparation, CCS cells were first washed with PBS and then lysed in radioimmunoprecipitation assay buffer supplemented with 1% protease/phosphatase inhibitor cocktail. Protein concentrations were measured using bicinchoninic acid (Thermo Fisher Scientific) according to the manufacturer's protocol. The cell lysates were separated on 4%–12% Bis-Tris gels (Life Technologies) and transferred to polyvinylidene difluoride membranes (Nippon Genetics), which were incubated in Tris-buffered saline (TBS) containing 5% skim milk and Tween 20 (TBS-T) at room temperature. The blocked membranes were incubated with primary antibodies (shown in Supplementary Table S1) in Can Get Signal Solution 1 (Toyobo) at 4°C overnight, after which they were incubated with secondary antibodies in Can Get Signal Solution 2 (Toyobo) at room temperature for 1 hour. After a wash with TBS-T, immunoreactive bands were visualized using chemiDOC touch (Bio-Rad).

### qRT-PCR Analysis

Total RNA was extracted using a RNeasy Mini Kit (catalog no. 74104; Qiagen) and reverse-transcribed to cDNA using ReverTra Ace qPCR RT Master Mix (Toyobo). Gene expression was measured using a StepOnePlus Real Time PCR System (Applied Biosystems) and SYBR Green Realtime PCR Master Mix (Toyobo). Target gene expression levels were normalized to the level of GAPDH. Relative expression was calculated using the 2^−ΔΔ*C*^_t_ method. The PCR primers (forward and reverse) used in this study are shown in Supplementary Table S1.

### siRNA Transfection

CCS cells were seeded at a density of 5 × 10^3^ cells/well in 96-well plates or 4 × 10^5^ cells/well in 6-well plates and cultured for 24 hours. The cells were then reverse-transfected with Lipofectamine RNAiMax (Invitrogen) according to the manufacturer's protocol using 5 nmol/L siRNAs targeting EWSR1::ATF1 and SOX10 as well as a nontargeting negative control siRNA purchased from Thermo Fisher Scientific (Supplementary Table S1).

### Plasmid Transfection for SOX10 Overexpression

CCS cells were seeded at a density of 3 × 10^6^ cells in 6-cm dish and cultured for 24 hours. The cells were then transfected with Lipofectamine 2000 (Invitrogen) according to the manufacturer's protocol using 8 μg plasmid pCMV6‐XL5‐SOX10 (OriGene). After 6 hours, growth medium was replaced.

### Reporter Gene Assays

In our reported gene assays, pNL1.2 (#N1001, Promega) was used as a vector. Fragment DNA was inserted into the pNL1.2 vector using Ligation high ver.2 (LGK-201, Toyobo) with *Kpn*I (#R0142S; New England Biolabs, Inc.) and *Nhe*I (#R0131S; New England Biolabs) according to the manufacturer's protocol. Transformation was performed using Competent Quick DH5a (DNA-913F; Toyobo), and plasmids were purified using NucleoBond Xtra Maxi (Macherey-Nagel) according to the manufacturer's instructions. Transfection was performed using Lipofectamine 2000 (Thermo Fisher Scientific) according to the manufacturer's protocol. Cells were assayed after vorinostat or vehicle treatment for 6 hours using the Nano-Glo Luciferase Assay System (N1120; Promega) with the GloMax Navigator System (Promega) according to the manufacturer's instructions. A measurement time of 1 second was used for NanoDLR, and the relative promoter activity was calculated.

### Cleavage Under Targets and Release Using Nuclease Assay

For the analysis of histone modification–promotor interactions, cleavage under targets and release using nuclease (CUT&RUN) assays were performed using the CUT&RUN assay Kit (#86652; Cell Signaling Technology) according to the manufacturer's protocol. CCS cells were seeded at 1 × 10^6^ cells per 6-cm dish and cultured for 24 hours, after which they were treated with 3 μmol/L vorinostat or vehicle. For each reaction, 1 × 10^5^ cells were used, and the cells were bound to concanavalin A beads and permeabilized with a digitonin-containing buffer. Antibodies were then applied (shown in Supplementary Table S1) at a dilution of 1:100 and incubated at 4°C overnight. Antibody-bound DNA was purified using DNA purification buffers and spin columns (14209S; Cell Signaling Technology) and amplified for use as templates in CUT&RUN–qRT-PCR or to construct libraries in CUT&RUN-sequencing (CUT&RUN-seq). One-hundred base pair paired-end sequencing was then performed using the NovaSeq 6000 System (Illumina). CUT&RUN-seq data were analyzed by mapping the reads using Bowtie2. The sequencing reads were aligned to human genome build hg38. The UCSC genome browser (12) was used to visualize the mapped reads.

### Single-cell Assay for Transposase-accessible Chromatin with High-throughput Sequencing and Single-cell RNA Sequencing

CCS cell lines were seeded at a density of 2 × 10^6^ per 10-cm dish and cultured for 24 hours, after which they were treated with 3 μmol/L vorinostat or vehicle. After 24 hours of treatment, the cells were washed twice with cold PBS + 0.04% BSA, and 1 × 10^6^ cells were used for nuclei isolation according to the manufacturer's protocol (version CG000365 Rev B). Briefly, the cells were spun at 300 rcf and 4°C for 5 minutes and then mixed with 100 μL of lysis buffer [prepared according to the instructions of 10x Genomics and containing 10 mmol/L Tris-HCl (pH 7.4), 10 mmol/L NaCl, 3 mmol/L MgCl_2_, 0.1% Tween-20, 0.1% Nonidet P40 Substitute, 0.01% digitonin, 1% BSA, 1 mmol/L dithiothreitol, 1 U/μL Roche RNase inhibitor, and water] and incubated on ice for 5 minutes. Subsequently, 1 mL of chilled wash buffer (prepared according to the manufacturer's protocol) was added before spinning. The cells were then washed three times with the wash buffer and mixed with chilled Diluted Nuclei Buffer (10 × Genomics). Gel Beads in emulsion (GEM) generation and single-cell libraries were provided according to the Chromium Next GEM Single-Cell Multiome ATAC + Gene Expression User Guide (CG000338 Rev B). In brief, following transposition, GEMs were generated by combining barcoded gel beads, transposed nuclei, a Master Mix including reverse transcription (RT) reagents, and partitioning oil on a Chromium Next GEM Chip J (PN-2000264; 10x Genomics). By incubating GEM in a thermal cycler at 37°C for 45 minutes and 25°C for 30 minutes, 10x-barcoded DNA [for the assay for transposase-accessible chromatin (ATAC)] from transposed DNA and 10x-barcoded full-length cDNA (for GEX) from polyadenylated mRNA were prepared. The reaction was stopped with a quenching step. Subsequently, the GEM was shredded, and the pooled fractions were obtained. First-strand cDNA was purified from the GEM-RT reaction mixture using silane magnetic beads. Libraries were sequenced on the Illumina NovaSeq 6000 system according to 10x Genomics settings, with a median depth of >50,000 reads per nucleus for the majority of samples. Data were analyzed using the R packages “Signac” and “Seurat.”

### Analysis of Differentially Expressed Genes

To determine the genes differentially expressed following drug administration, the FindMarkers tool based on a nonparametric Wilcoxon rank sum test from Seurat was used. The adjusted *P* value was set as <0.05 and the log2 [fold change (FC)] was ≥ 0.5. Differentially expressed gene (DEG) data are available in Supplementary Table S2.

### Gene Ontology Analysis

Gene Ontology (GO) analysis was performed using the Database for Annotation, Visualization and Integrated Discovery (DAVID) online database v6.8 (https://david.ncifcrf.gov/) (13, 14). GO analysis was conducted with the species set as *Homo sapiens*, identifier set as the official gene symbol, gene list set as the list type, and remaining parameters set as the default values. The results comprised GO Biological Process (BP) terms. GO data are available in Supplementary Tables S3 and S4.

### Subcutaneous Xenograft Assays

All animal experiments were performed using protocols approved by the Institutional Animal Care and Use Committee of the Osaka University Graduate School of Medicine (Osaka, Japan). Five-week-old female BALB/c nu/nu mice (SLC) were used. For the subcutaneous xenograft assays, a subcutaneous injection of 1 × 10^7^ CCS cells was administered to the left side of the back of individual mice. Tumor size was measured twice per week using a caliper, and tumor volume was calculated using the following formula: size = length × width × width/2. Treatment began when all tumors became palpable. Prior to treatment, mice were randomly assigned to treatment cohorts. Vorinostat was administrated at 50 or 20 mg/kg by intraperitoneal injection once daily. Mivebresib was administrated at 0.4 mg/kg by oral gavage once daily. In combination therapy, 20 mg/kg vorinostat and 0.4 mg/kg mivebresib were administrated. As a control, an equal volume of DMSO was administrated by intraperitoneal injection and/or oral gavage at the same time. Xenograft tumor volume and mouse body weight were measured twice per week. The mice were continually monitored for marked adverse effects, and tumors were collected and weighed at the end of the treatment period.

### Statistical Analysis

All data are expressed as means ± SDs. Either a two-tailed Student *t* test or Wilcoxon signed-rank test was used to determine statistical differences. *P* values of <0.05 were considered statistically significant, and the specific *P* values are indicated in the figure legends.

### Data Availability

The data generated in this study are publicly available in the National Center for Biotechnology Information at accession IDs PRJNA972279, PRJNA972291. The other data generated are available upon request from the corresponding author.

## Results

### High-throughput Screening Identifies Vorinostat as a Potential Therapeutic Agent Against CCS

To identify effective therapeutic agents against CCS, we tested 1,134 FDA-approved drugs at a fixed concentration (10 μmol/L) using a high-throughput screening method with Hewga-CCS cells. Almost all tested drugs were ineffective against CCS (Supplementary Fig. S1A), but 26 drugs resulted in a cell viability of <30% (Supplementary Fig. S1B). We excluded conventional cytotoxic agents, tyrosine kinase inhibitors, dyeing agents, and disinfectants from the 26 drugs, subjecting the remaining agents to a dose-dependent cell viability assay. Proteasome inhibitors did not exhibit dose dependency; therefore, we selected the HDACi vorinostat for further study. Among the 1,134 tested drugs, only one HDACi was identified.

### Antiproliferative Effect of Vorinostat on CCS Cells

**FIGURE 1:**
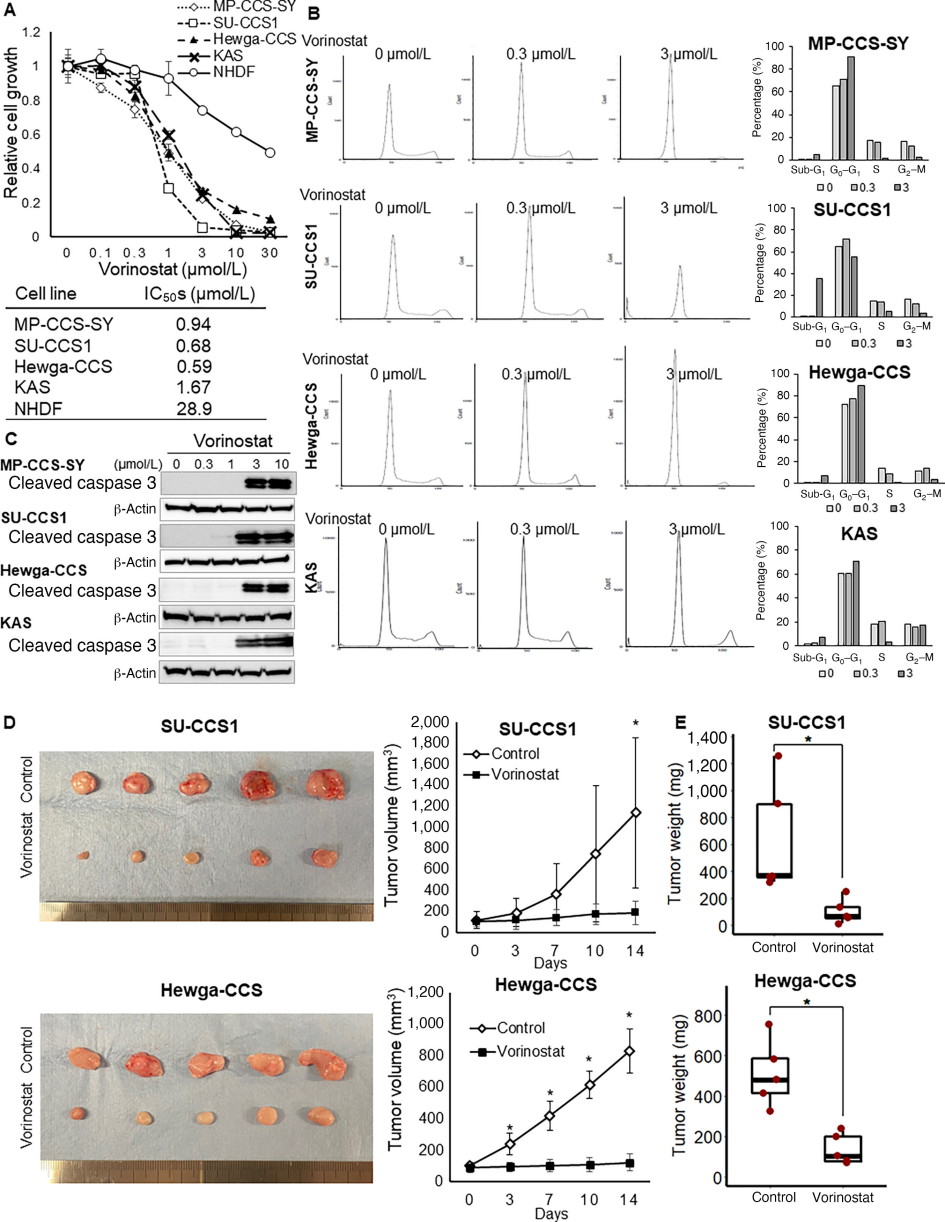
Vorinostat treatment inhibits CCS cell growth *in vitro* and *in vivo*. **A,** MP-CCS-SY, SU-CCS1, Hewga-CCS, KAS, and NHDF cells were incubated with 0–30 μmol/L vorinostat for 48 hours, and the viable number of cells were estimated via a WST-8 assay (*n* = 3). The calculated IC_50_ values are shown in Table 1. **B,** Four CCS cell lines were treated with 0.3 and 3 μmol/L vorinostat or vehicle for 48 hours, stained with PI, and analyzed for cell-cycle stage using flow cytometry. **C,** Four CCS cell lines were treated with 0.3 to 10 μmol/L vorinostat or vehicle for 24 hours, and the protein expression levels of the apoptosis markers cleaved caspase-3 was detected using Western blotting. **D** and **E,** SU-CCS1 and Hewga-CCS cells were engrafted in nude mice, which were treated with 50 mg/kg vorinostat or vehicle via intraperitoneal administration (5 mice/group). Volume (D) and weight (E) of SU-CCS1 and Hewga-CCS tumors during treatment are shown (*n* = 5). Data in A, D, and E are means ± SDs. *, *P* < 0.05; **, *P* < 0.01 (Student *t* test).

To investigate the antitumor effects of vorinostat on CCS, we performed a cell viability assay using four CCS and NHDF cell lines. Vorinostat reduced the number of viable cells in a dose-dependent manner in all four cell lines. CCSs were more sensitive than NHDFs, with 50% inhibitory concentration (IC_50_) values of 0.59–1.67 μmol/L and 28.9 μmol/L, respectively (Fig. 1A). Among the examined STS cell lines, CCS and Ewing sarcoma cell lines exhibited higher sensitivity to vorinostat (Table 1). Interestingly, both subtypes harbor the *EWSR1* gene as a fusion gene partner (*EWSR1::ATF1* and *EWSR1::FLI1*). In cell-cycle analyses, vorinostat treatment increased the G_0_–G_1_ or sub-G_1_ fraction in all four CCS cell lines (Fig. 1B). In addition, the cleaved caspase-3 protein was detected in the four CCS cell lines following vorinostat treatment (Fig. 1C). These results indicate that vorinostat reduces CCS cell proliferation by inducing G_0_–G_1_ cell-cycle arrest and apoptosis *in vitro*. We also examined the antitumor effects of vorinostat on CCS cells *in vivo*. Compared with the vehicle control, daily treatment with vorinostat significantly reduced tumor volume and weight in both xenograft models (Fig. 1D and E). Thus, vorinostat acts as an active agent in CCS cells.

**TABLE 1:** IC_50_s of various tumor cell lines

Cell line	IC_50_ (μmol/L)
Clear cell sarcoma	
MP-CCS-SY	0.94
SU-CCS1	0.68
Hewga-CCS	0.69
KAS	1.35
Ewing sarcoma	
EW8	0.87
Synovial sarcoma	
Aska-SS	2.15
HS-SY-II	1.71
SYO-1	1.99
Yamato-SS	1.73
Fibrosarcoma	
HT1080	2.99
Liposarcoma	
SW872	1.76
Epithelioid sarcoma	
Asra-EPS	11.4
VAESBJ	15.1
NEPS	16.6
Normal human dermal fibroblasts	
NHDF	28.9

### Vorinostat Suppresses *EWSR1::ATF1* Expression in CCS Cells

**FIGURE 2:**
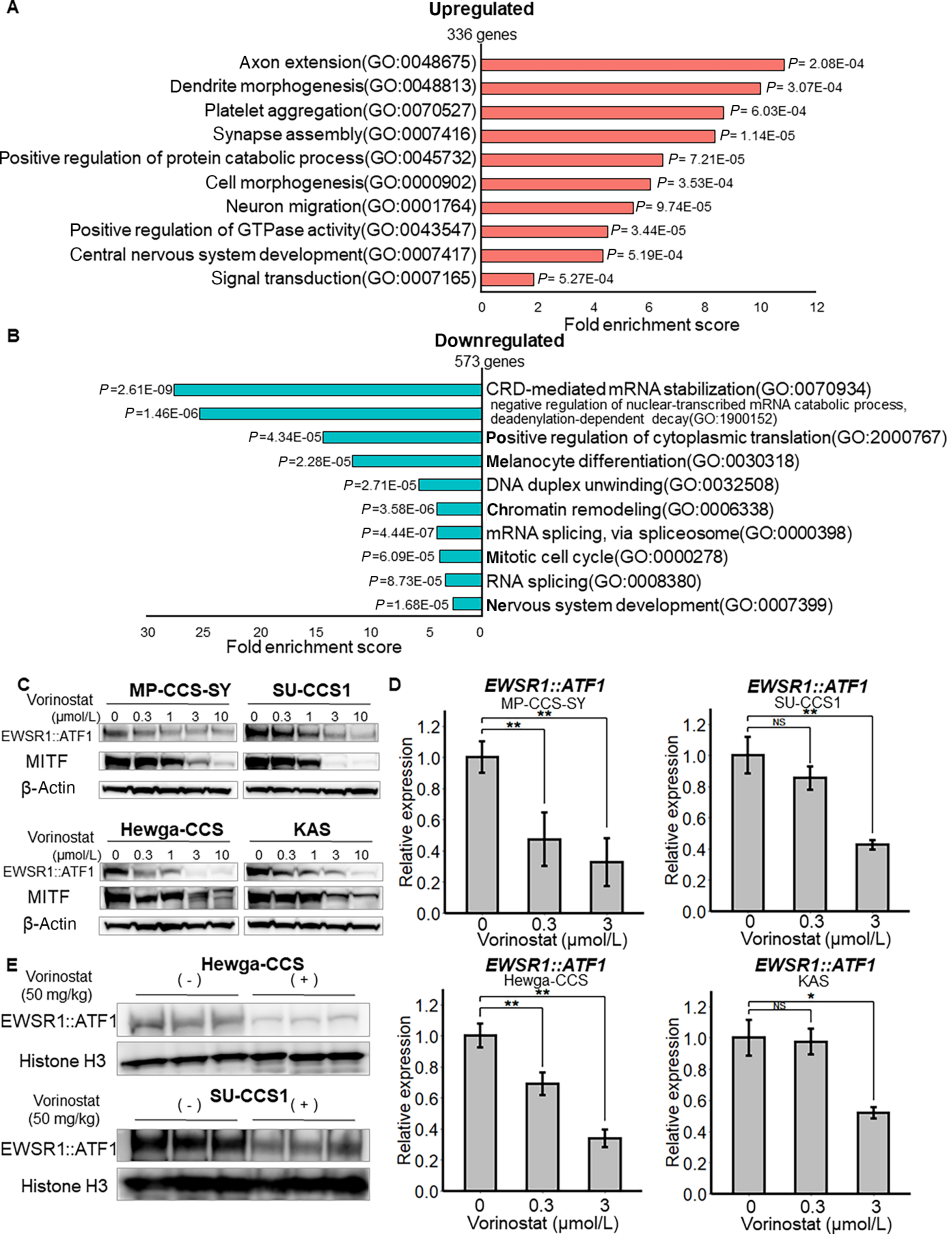
Vorinostat regulates the expression of EWSR1::ATF1*.***A** and **B,** Ten terms with the lowest *P* values in GO analysis are listed in FC order. The most significantly upregulated (A) and downregulated (B) biological terms are indicated. **C,** CCS cells were treated with 0, 0.3, 1.0, 3.0, or 10.0 μmol/L vorinostat for 24 hours. EWSR1::ATF1 and MITF protein expression levels were estimated via Western blotting. **D,** CCS cells were treated with 0, 0.3, or 3.0 μmol/L vorinostat for 24 hours. *EWSR1::ATF1* mRNA levels in CCS cells were quantified using qRT-PCR (normalized to GAPDH; *n* = 3). **E,** Tumor tissues were collected from SU-CCS1 and Hewga-CCS xenografts at the endpoint. EWSR1::ATF1 protein expression levels were analyzed via Western blotting (*n* = 3). Data in D, means ± SDs. *, *P* < 0.05; **, *P* < 0.01 (Student *t* tests).

DEG analysis of scRNA-seq data revealed that 336 and 573 genes were upregulated and downregulated, respectively (Supplementary Tables S3 and S4). GO analysis indicated that the most significantly upregulated and downregulated BP terms were related to the development of the nervous system (Fig. 2A) and to mRNA transcription and melanocyte differentiation (Fig. 2B), respectively. Next, we examined the effect of vorinostat on the tumor-specific fusion oncogene *EWSR1::ATF1* and its downstream microphthalmia-associated transcription factor (MITF; ref. 5). Vorinostat reduced the protein levels of both EWSR1::ATF1 and MITF in a dose-dependent manner in all four CCS cell lines (Fig. 2C). The mRNA expression of *EWSR1::ATF1* was also significantly suppressed by vorinostat treatment in these four CCS cell lines (Fig. 2C). Consistent with a previous study (9), romidepsin, another HDACi, reduced *EWSR1::ATF1* mRNA expression in MP-CCS-SY and SU-CCS1 cells (Supplementary Fig. S2A). In addition, scRNA-seq analysis results indicated that vorinostat significantly decreased the expression levels of *EWSR1::ATF1* in MP-CCS-SY cells (Supplementary Fig. S2B). To determine the silencing effects of *EWSR1::ATF1* on cell growth, we examined CCS cell proliferation using two types of siRNA (validation data are shown in Supplementary Fig. S2C and S2D). *EWSR1::ATF1* knockdown significantly inhibited the growth of MP-CCS-SY and SU-CCS1 cells (Supplementary Fig. S2E). We also confirmed fusion gene suppression following HDACi treatment in xenografted tumors (Fig. 2E). These results suggest that HDACi is a promising drug for targeting the CCS-specific fusion oncogene *EWSR1::ATF1*.

### Vorinostat Treatment Reduces Both H3K27ac and H3K9ac Levels at the EWSR1 Promoter Region

**FIGURE 3:**
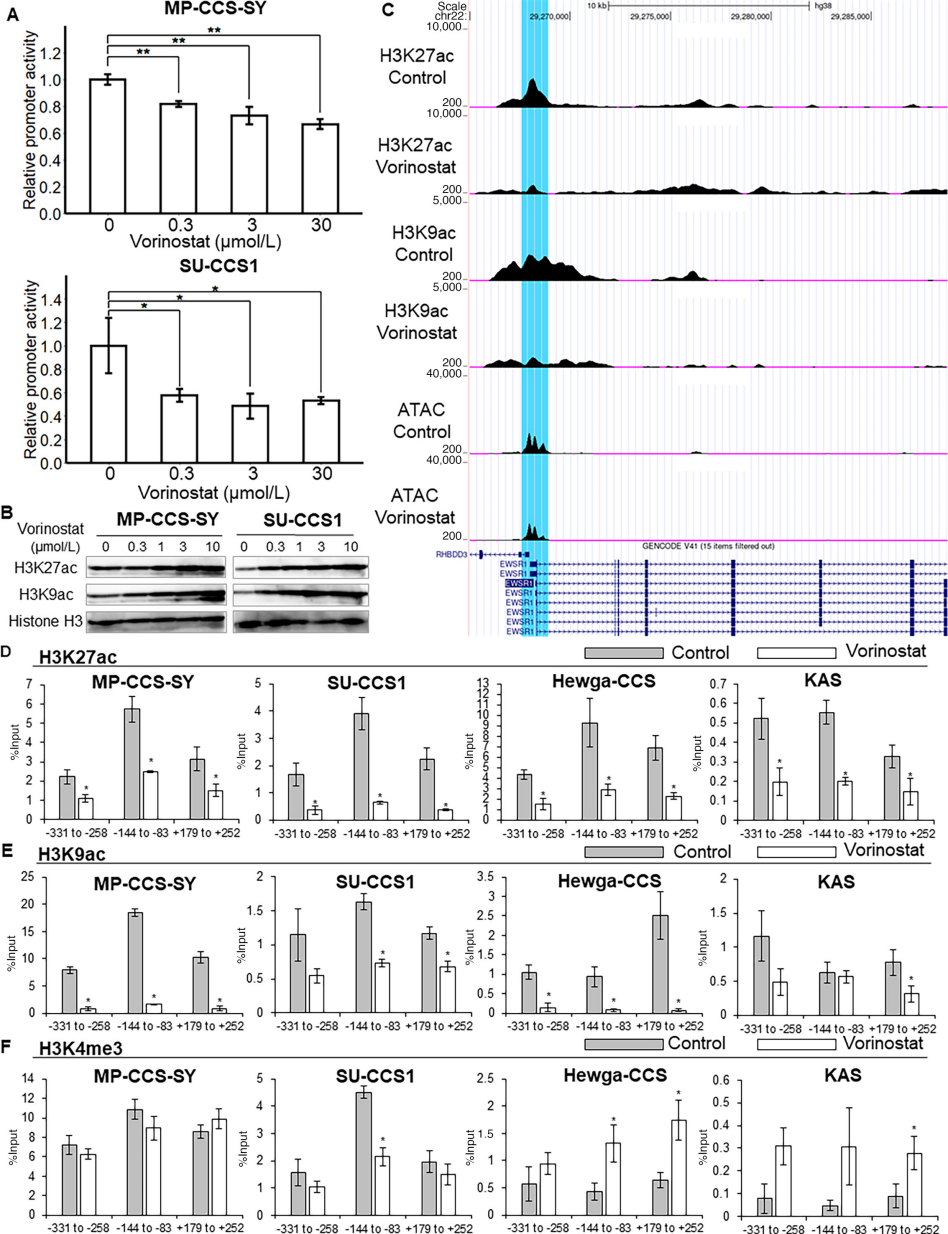
Vorinostat alters EWSR1 promoter activity and histone modification at the promoter region. **A,** CCS cells were treated with 0, 0.3, 3.0, or 30.0 μmol/L vorinostat for 6 hours. Relative EWSR1 promoter activity was measured via a reporter assay (*n* = 3). **B,** MP-CCS-SY and SU-CCS1 cells were treated with 0–10 μmol/L vorinostat for 24 hours, and the protein expression levels of the active acetyl-histone markers H3K27ac and H3K9ac were detected using Western blotting. **C,** Genome browser screenshot of the epigenome maps at EWSR1. Tracks visualize CUT&RUN-seq data for two acetyl-histone markers (H3K27ac and K3K9ac) (*n* = 2), and scATAC-seq data are shown for treatments with 3 μmol/L vorinostat or vehicle for 24 hours. Blue highlights indicate the inserted DNA regions used in the reporter assay. Four CCS cells were treated with 3 μmol/L vorinostat or vehicle for 24 hours, and the levels of H3K27ac (**D**), H3K9ac (**E**), and H3K4 me3 (**F**) at three sites within the EWSR1 promoter region were analyzed using CUT&RUN-qPCR (*n* = 3). The values shown are relative to the input. Data in A and D–F are means ± SDs. *, *P* < 0.05; **, *P* < 0.01 (Student *t* test).

To elucidate the mechanism underlying HDACi-mediated EWSR1::ATF1 transcriptional repression in CCS cell lines, we investigated the effect of vorinostat on EWSR1 promoter activity. A reporter assay was performed using a luciferase reporter construct containing the EWSR1 promoter region: vorinostat treatment significantly downregulated EWSR1 promoter activity in MP-CCS-SY and SU-CCS1 cells (Fig. 3A). We also investigated the effect of vorinostat on the histone modification of the promoter region according to the statuses of H3K27ac, H3K9ac, and H3K4me3, which are reported to be enriched at the EWSR1 promoter region and are associated with *EWSR1::FLI1*–dependent gene expression (15, 16). Vorinostat increased the total acetylation of H3K27 and H3K9 in a dose-dependent manner while decreasing the levels of both H3K27ac and H3K9ac at the EWSR1 promoter region in MP-CCS-SY cells (Fig. 3B and C). CUT&RUN-qPCR analysis results also revealed that vorinostat significantly decreased the enrichment of H3K27ac and H3K9ac at the EWSR1 promoter region in all four CCS cell lines (Fig. 3D and E). Although H3K4me levels at the EWSR1 promoter region were unchanged in MP-CCS-SY and SU-CCS1 cells, these levels were increased in Hewga-CCS and KAS cells (Fig. 3F). To determine whether chromatin structure was altered by vorinostat treatment, we performed a single-cell ATAC with high-throughput sequencing (scATAC-seq) in MP-CCS-SY cells. Although vorinostat treatment reduced the enrichment of H3K27ac and H3K9ac at the EWSR1 promoter region, a moderate decline in ATAC-seq was observed in the region (Fig. 3C). These results suggest that vorinostat decreases *EWSR1::ATF1* expression levels by reducing EWSR1 promoter activity and H3K27ac and H3K9ac levels in its promoter region as well as moderately altering chromatin structure.

### Reduction of BRD4 Levels at the EWSR1 Promoter Region Suppresses *EWSR1::ATF1* Transcription

**FIGURE 4:**
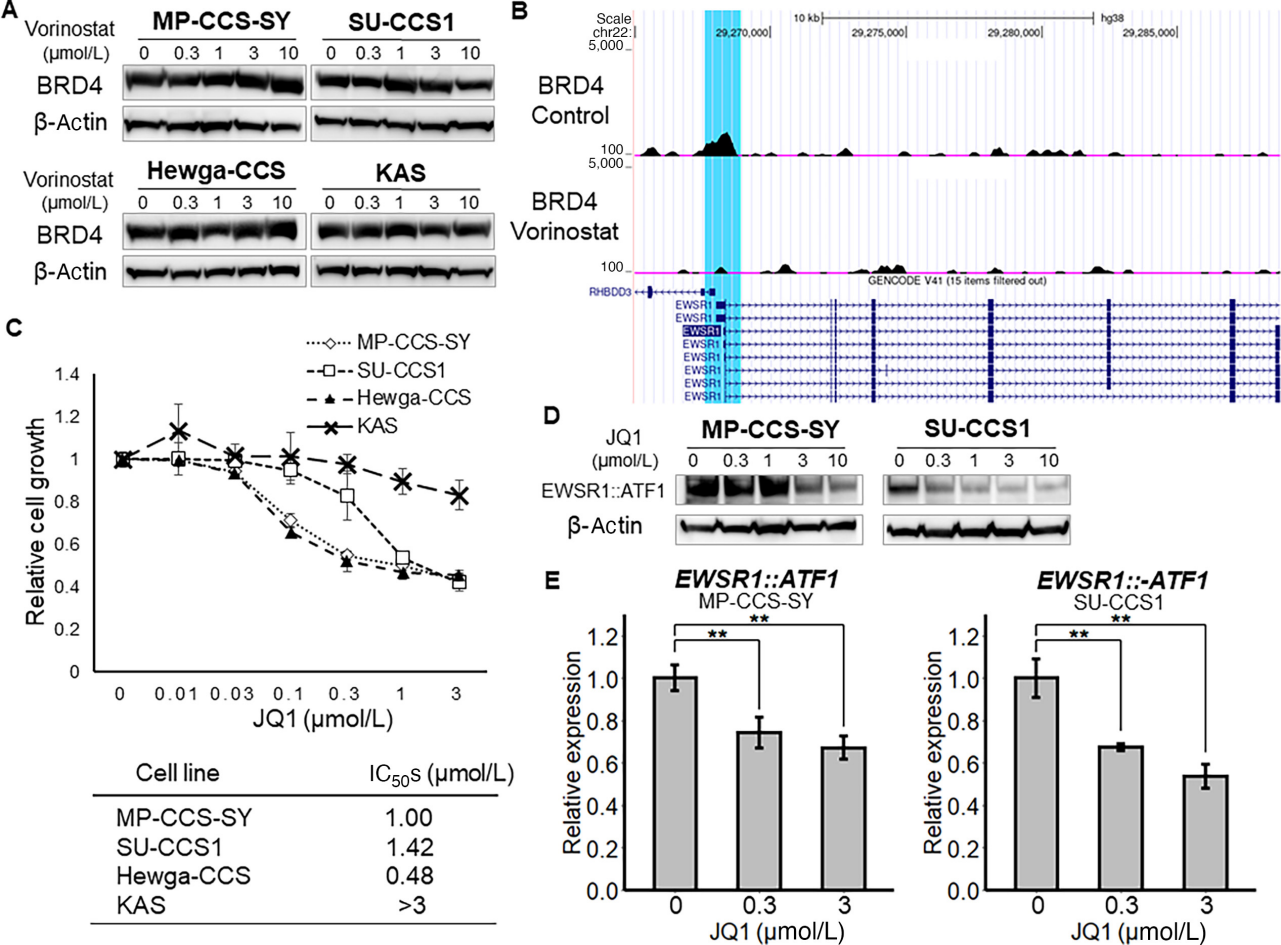
Depletion of BRD4 from the EWSR1 promoter region suppresses the transcription of EWSR1::ATF1*.***A,** CCS cells were treated with 0–10 μmol/L vorinostat for 24 hours, and the protein expression of BRD4 was detected using Western blotting. **B,** Genome browser screenshot of the epigenome maps at EWSR1. Tracks visualize CUT&RUN-seq data for BRD4 (*n* = 2), and scATAC-seq data are shown for treatment with 3 μmol/L vorinostat or vehicle for 24 hours. Blue highlight shows the EWSR1 promoter region. **C,** CCS cells were treated with 0–3 μmol/L JQ1 for 48 hours, and the number of viable cells was estimated using a WST-8 assay (*n* = 3). The calculated IC_50_ values are shown in Table 1. **D,** CCS cells were treated with 0, 0.3, 1.0, 3.0, or 10.0 μmol/L JQ1 for 24 hours. EWSR1::ATF1 protein expression levels were estimated via Western blotting. **E,** CCS cells were treated with 0, 0.3, and 3.0 μmol/L JQ1 for 24 hours. *EWSR1::ATF1* mRNA levels in CCS cells were quantified using qRT-PCR (normalized to GAPDH; *n* = 3). Data in C and E are means ± SDs. *, *P* < 0.05; **, *P* < 0.01 (Student *t* test).

The relatively small change of ATAC-seq in the EWSR1 promoter region compared with that of H3K27ac and H3K9ac prompted us to explore other mechanisms of transcriptional regulation through histone acetylation. Accordingly, we investigated BRD4, a member of the bromodomain and extraterminal (BET) protein family, which accumulates on acetylated chromatin regions, functioning as a nucleation center for the assembly of large protein complexes that promote RNA polymerase II activity, which stimulates transcription initiation and elongation (17, 18). Several studies have reported that JQ1, a BET inhibitor (BETi), is effective in sarcoma cell lines (19, 20). Vorinostat treatment did not alter the total amount of BRD4 in CCS cells but lowered BRD4 binding at the EWSR1 promoter region in MP-CCS-SY cells (Fig. 4A and B), implying that reduced H3K27ac and H3K9ac levels at the promoter region lead to a decrease in BRD4 recruitment and the suppression of *EWSR1::ATF1* transcription. To determine the effects of BRD4 on *EWSR1::ATF1* expression and CCS cell proliferation, we treated CCS cell lines with JQ1 finding that JQ1 reduced the number of viable cells in four cell lines in a dose-dependent manner (Fig. 4C). MP-CCS-SY, SU-CCS1, and Hewga-CCS cells were the most sensitive cell lines, exhibiting IC_50_ values of 0.48–1.42 μmol/L, whereas KAS cells were the least sensitive. In addition, mivebresib (ABBV-075), another BETi, also showed a dose-dependent inhibition of cell viability against three CCS cell lines, exhibiting IC_50_ values of 0.67–1.68 μmol/L (Supplementary Fig. S3A). JQ1 and mivebresib treatment also reduced the protein and mRNA levels of *EWSR1::ATF1* in a dose-dependent manner in MP-CCS-SY and SU-CCS1 cells (Fig. 4D and E; Supplementary Fig. S3B). These results suggest that a vorinostat-induced reduction in histone acetylation at the EWSR1 promoter region affects EWSR1::ATF1 expression at least partly through the modulation of BRD4 recruitment.

### SOX10 Regulates the Expression of EWSR1::ATF1

**FIGURE 5:**
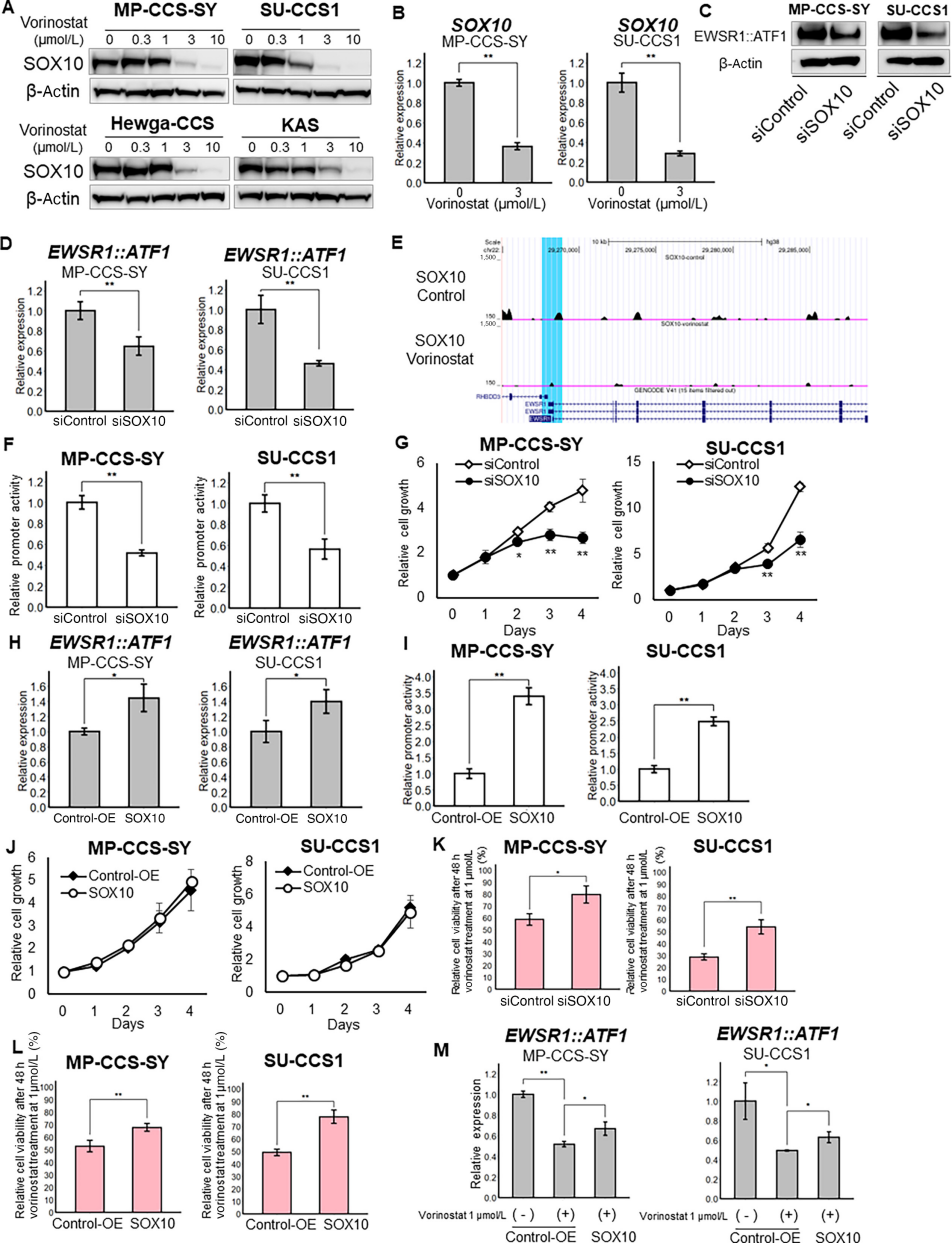
SOX10 regulates the expression of EWSR1::ATF1. **A,** CCS cells were treated with 0, 0.3, 1.0, 3.0, or 10.0 μmol/L vorinostat for 24 hours. SOX10 protein expression levels were estimated via Western blotting. Loading controls are identical to those shown in Fig. 4A. **B,** CCS cells were treated with 0 or 3 μmol/L vorinostat for 24 hours. *SOX10* mRNA expression levels in CCS cells were quantified using qPCR (normalized to GAPDH; *n* = 3). **C,** EWSR1::ATF1 protein expression levels of MP-CCS-SY and SU-CCS1 cells 48 hours after SOX10 knockdown were detected via Western blotting. **D,***EWSR1::ATF1* mRNA expression levels of MP-CCS-SY and SU-CCS1 cells 48 hours after SOX10 knockdown were quantified using qRT-PCR (normalized to GAPDH; *n* = 3). **E,** Genome browser screenshot of the epigenome maps at EWSR1. Tracks visualize CUT&RUN-seq data for SOX10 (*n* = 2), and scATAC-seq data are shown for treatments with 3 μmol/L vorinostat or vehicle for 24 hours. Blue highlights indicate the inserted DNA regions used in the reporter assay. **F,** Relative EWSR1 promoter activity of MP-CCS-SY and SU-CCS1 cells 48 hours after SOX10 knockdown was measured with a reporter assay (*n* = 3). **G,** Proliferation of CCS cells after SOX10 knockdown was measured using a WST-8 assay during 1–4 days of culture. **H,** EWSR1::ATF1 mRNA expression levels of MP-CCS-SY and SU-CCS1 cells 48 hours after SOX10 overexpression. **I,** Relative EWSR1 promoter activity of MP-CCS-SY and SU-CCS1 cells 24 hours after SOX10 overexpression was measured with a reporter assay (*n* = 3). **J,** Proliferation of CCS cells after SOX10 overexpression was measured using a WST-8 assay during 1–4 days of culture. **K,** MP-CCS-SY and SU-CCS1 cells after SOX10 knockdown or not were treated with vorinostat at 1 μmol/L. Relative cell viability was assessed after 48 hours by WST-8 assay. **L,** MP-CCS-SY and SU-CCS1 cells after SOX10 overexpression or not were treated with vorinostat at 1 μmol/L. Relative cell viability was assessed after 48 hours by WST-8 assay. **M,** MP-CCS-SY and SU-CCS1 cells after SOX10 overexpression or not were treated with vorinostat at 1 μmol/L. Relative *EWSR1::ATF1* mRNA expression levels were quantified using qRT-PCR (normalized to GAPDH; *n* = 3). Data in B, D, F–M are means ± SDs. *, *P* < 0.05; **, *P* < 0.01; ns, nonsignificant (Student *t* test).

To determine the transcription factors regulating EWSR1::ATF1 promoter activity, we used the FindMotifs tool from Signac. All motifs contained in the significantly higher ATAC peaks of the control group relative to those of the vorinostat group are listed in Supplementary Table S5. Because SOX10 exhibited the highest fold enrichment with the lowest *P* value among all 746 motifs, we examined the effect of SOX10 on EWSR1::ATF1 expression. First, we investigated SOX10 protein expression during vorinostat treatment, finding a dose-dependent reduction in this expression (Fig. 5A). In addition, the mRNA expression of *SOX10* was suppressed by vorinostat treatment (Fig. 5B), and our scRNA-seq analysis results were consistent with this finding (Supplementary Fig. S4A). The same results were obtained using romidepsin treatment (Supplementary Fig. S4B). Next, we evaluated the silencing effect of SOX10 on EWSR1::ATF1 expression. Interestingly, the knockdown of *SOX10* reduced the expression of EWSR1::ATF1 at both the protein and mRNA levels (Fig. 5C and D; siRNA validation data are shown in Supplementary Fig. S4C and S4D). However, the knockdown of *EWSR1::ATF1* did not reduce SOX10 expression (Supplementary Fig. S4E). To confirm the transcriptional control activity of SOX10 toward EWSR1::ATF1, we conducted CUT&RUN-seq and a reporter assay. CUT&RUN-seq for SOX10 revealed that vorinostat reduced the enrichment of SOX10 at the EWSR1 promoter region in MP-CCS-SY cells (Fig. 5E, blue highlighted region). A promoter assay indicated that *SOX10* knockdown lowered EWSR1 promoter activity (Fig. 5F). Furthermore, a WST-8 assay revealed that *SOX10* knockdown significantly inhibited the growth of MP-CCS-SY and SU-CCS1 cells (Fig. 5G). In contrast, *SOX10* overexpression (validation data are shown in Supplementary Fig. S4F) increased *EWSR1::ATF1* mRNA expression and its promoter activity, but did not promote cell proliferation (Fig. 5H–J). To determine whether knockdown or overexpression of SOX10 could suppress the antiproliferation activity of vorinostat, we performed the WST-8 assay. As expected, both knockdown and overexpression of *SOX10* reduced the drug efficacy of vorinostat on CCS cells due to the loss of SOX10-targeting effects (Fig. 5K and L). We also confirmed that *SOX10* overexpression partially rescued the downregulation of EWSR1::ATF1 by vorinostat treatment (Fig. 5M). These results suggest that SOX10 controls *EWSR1::ATF1* transcription in CCS cells, and SOX10 regulation is one of the mechanisms underlying vorinostat-dependent EWSR1::ATF1 suppression.

### A HDACi and BETi Combination is an Effective Therapeutic Option for CCS

**FIGURE 6:**
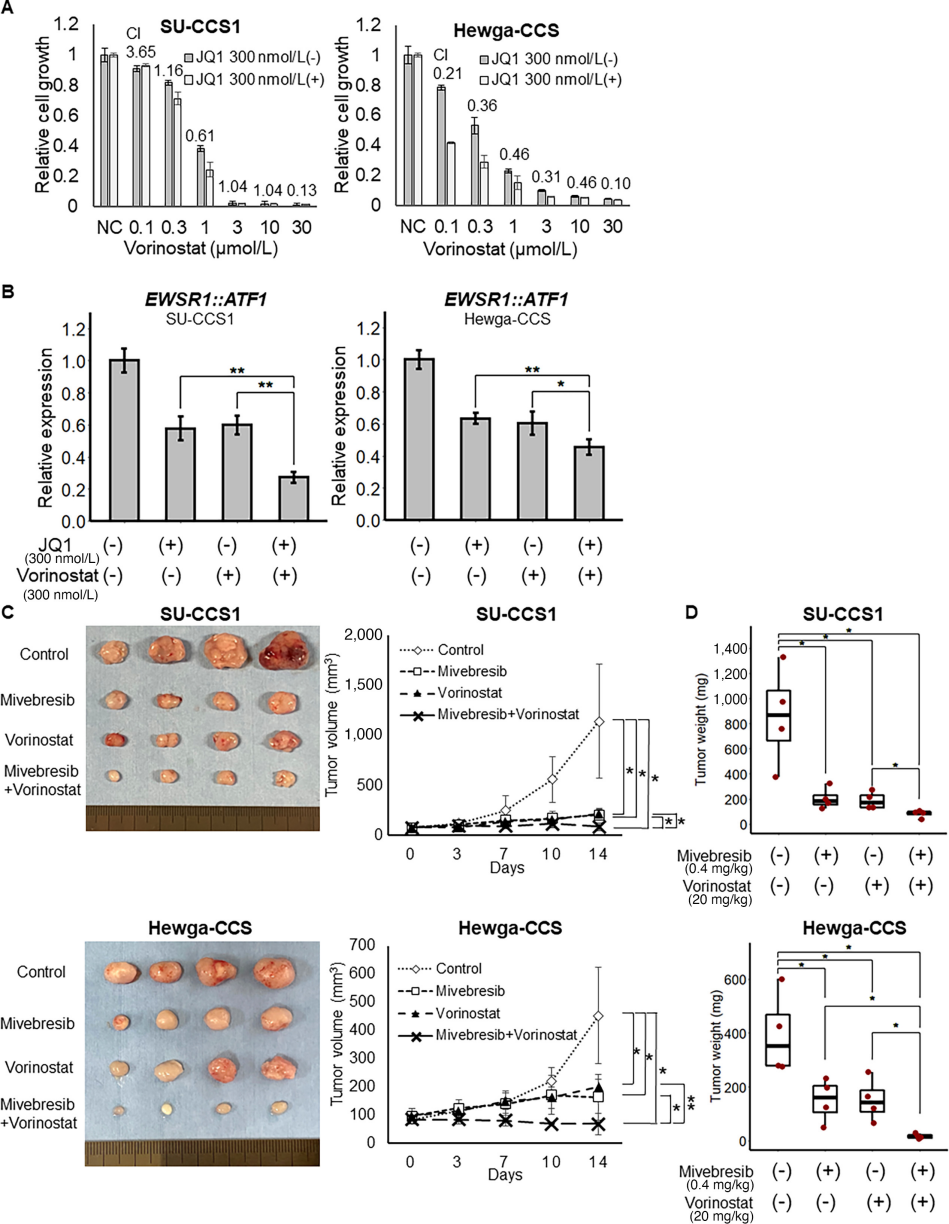
BET inhibitors enhance the effect of vorinostat on CCS. **A,** SU-CCS1 and Hewga-CCS cells were treated for 48 hours with the indicated concentrations of vorinostat in the absence (–) or presence (+) of 300 nmol/L JQ1, and the viable number of cells was estimated using a WST-8 assay (*n* = 3). The calculated combination index values are shown in the histogram. **B,** SU-CCS1, and Hewga-CCS cells were treated for 24 hours with the absence (–) or presence (+) of 300 nmol/L vorinostat or JQ1. *EWSR1::ATF1* mRNA levels in CCS cells were quantified using qRT-PCR (normalized to GAPDH; *n* = 3). **C** and **D,** SU-CCS1 and Hewga-CCS cells were engrafted in nude mice, which were treated with vehicle (controls), or 0.4 mg/kg mivebresib, or 20 mg/kg vorinostat, or both drugs in combination (4 mice/group). Tumor volume (C) and weight (D) of SU-CCS1 and Hewga-CCS tumors during treatment are shown (*n* = 4). *, *P* < 0.05; **, *P* < 0.01 (Student *t* test).

Finally, we explored the effect of combination therapy using a HDACi and BETi on CCS cell lines. Compared with single agent treatments, the combined treatment of vorinostat and JQ1 or mivebresib resulted in a sharp dose-dependent reduction in relative cell viability, and the combination index calculated using the Chou method was <1 at 1 μmol/L vorinostat (Fig. 6A, Supplementary Fig S5A). In addition, we examined the effects of the combination therapy on *EWSR1::ATF1* expression. Compared with single agent treatments, a combined treatment of vorinostat and JQ1 led to significantly higher suppression of *EWSR1::ATF1* mRNA (Fig. 6B). We also examined the effects of the combination therapy on CCS cells *in vivo*. Compared with the single agent groups, the combination of vorinostat and mivebresib group significantly decreased tumor growth (Fig. 6C and D). These results suggest that a combined HDACi and BETi treatment might be a potential therapeutic option for CCS.

## Discussion

The development of new drugs for CCS is challenging due to the rarity of the disease. Large-scale clinical genomic profiling analyses showed that 13.5% of CCS has c-MYC amplification and <20% of CCS has actionable mutations higher than level 4, as defined by OncoKB (http://oncokb.org; ref. 21). Other large-scale clinical sequencing analyses showed that <20% of CCS harbors TERT promoter mutations and that the level of genetic similarity among CCSs is relatively low (22). Therefore, a genome-informed approach to CCS therapy is challenging. We exposed a CCS cell line to FDA-approved drugs, thereby identifying the HDACi vorinostat as a potentially active agent against CCS. To date, several preclinical studies showed that vorinostat is effective in sarcoma (23, 24), and three phase II trials assessing the effects of HDACis against STSs have been conducted (25–27); however, the results were disappointing, and no partial or complete response was observed in any patient. However, among the 109 patients enrolled in the 3 studies, only 1 patient with CCS was included. Therefore, we believe that HDACis could be used as therapeutic agents against CCS. However, considering the low response rate of HDACis in other patients with STS, combination therapy with HDACis should be investigated as a possible solution.

HDACis have been reported to exert antitumor effects by upregulating tumor suppressor genes (28, 29), inducing apoptosis (30), decreasing invasion (31), decreasing metastasis (32), and regulating autophagy (33). Regarding CCS, cell-cycle arrest, apoptosis, and decreased EWSR1::ATF1 expression were observed in previous cell line experiments (10). The direct inhibition of disease-specific fusion oncogenes or their signals is a possible method for overcoming CSS; however, owing to the unknown oncogenic mechanism and the multitargetable potential of fusion oncogenes, such methods have only been established in cases where the fusion component is associated with specific targetable genes such as in *BCR-ABL* (34), ALK-/ROS-/RET-rearrangement lung cancer (35–37), and NTRK-rearrangement solid tumors (38). Consistent with a previous study, we found that HDACi treatment decreases the expression of EWSR1::ATF1 in CCS cells, and we focused on elucidating the underlying suppression mechanism, which would facilitate the development of fusion gene–targeted therapy. Unlike the transcriptional activation mechanisms of HDACis, the transcriptional suppression mechanisms of HDACis are not well understood. Histone deacetylation around the transcription start site of the suppressed gene, decreased binding of transcription factors or histone acetyl transferase at deacetylated regions, and the blocking of RNA polymerase II elongation were reported previously as possible suppression mechanisms (39–41). Whether these effects are the primary or secondary events of HDACi treatment is still unclear (40, 41). However, consistent with the previous studies, we demonstrated deacetylation and decreased transcriptional factor binding at the EWSR1::ATF1 promoter region. One previous study showed that HDACi alters BRD4-binding targets via H4 polyacetylation and upregulates transcription at BRD4-binding sites (42). Interestingly, following HDACi treatment, we observed a reduction in BRD4 binding at the EWSR1::ATF1 promoter region. These findings suggest that a similar mechanism of BRD4 retargeting is evoked during the HDACi-induced downregulation of gene expression.

SOX10 is expressed in neural crest stem cells and their melanoblastic and glial derivatives, and it regulates neural crest development and determines cell fate (43, 44). CCS was reported to originate from neural crest cells and to express SOX10 (5). Furthermore, although EWSR1::ATF1 is associated with multiple tumors, including angiomatoid fibrous histiocytoma, myoepithelial tumors, hyalinizing clear cell carcinoma, and CCS-like tumors of the gastrointestinal tract (45, 46), SOX10 expression is relatively specific to CCS (45, 47, 48). The role of SOX10 in CCS has not been clarified; however, ChIP-seq analysis revealed that SOX10 and EWSR1::ATF1 share 56%–83% of their binding site and cooperatively regulate MITF expression (49, 50). Consequently, we believe that SOX10 plays a critical role in the malignant behavior of CCS. Indeed, we demonstrated that SOX10 knockdown attenuated CCS proliferation. Interestingly, overexpression of SOX10 did not increase cell proliferation (Fig. 5E). A possible explanation is that SOX10 expression is already abundant in CCS cells, so additional SOX10 expression could attenuate vorinostat efficacy, but excess SOX10 beyond necessity could not increase the inherent malignant potential of CCS cells. Importantly, we found that SOX10 regulates the expression of EWSR1::ATF1. To our knowledge, this is the first study to identify the transcription factors that regulate fusion oncogene expression in sarcomas. Taken together, our findings suggest that SOX10 plays a crucial role in CCS biology.

On the basis of aforementioned results, we explored a combination therapy including a HDACi to achieve the downregulation of EWSR1::ATF1. Theoretically, HDACis enhance BRD4 binding; thus, we might predict that BETis would exert the opposite effects to HDACis. However, several studies have shown that HDACis and BETis exert synergistic antitumor effects, including in sarcoma cell lines (51–54). Little is known about the synergistic mechanism, but the HDACi-induced alteration of the BRD4 target partially mimics the effect of BETis (42). We found that a HDACi reduced BRD4 binding at the promoter region of EWSR1::ATF1 and that a BETi enhanced gene suppression. Thus, our findings support previous speculation and provide the rationale for the synergistic antitumor effects of HDACi and BETi combination therapy. Such a combination is also promising regarding fusion gene targeted therapy. Phase I/II clinical trial of HDACi and BETi combination therapy is now recruiting (NCT05053971), and we hope for encouraging results from the clinical trial.

In summary, we identified the HDACi vorinostat as an active agent for CCS therapy. This HDACi suppressed the expression of the *EWSR1::ATF1* fusion oncogene, and our study revealed the epigenetic and transcriptional mechanisms underlying this effect. For the first time, we identified a transcription factor that regulates EWSR1::ATF1 expression. Furthermore, we found a combination therapy that enhances EWSR1::ATF1 suppression. Collectively, our findings provide a blueprint for fusion gene–targeted therapy and a potential new therapy for CCS.

## Supplementary Material

Supplementary Tables S1-S5 Supplementary Table S1;List of resources (antibodies, primers, siRNA).
Supplementary Table S2;DEG analysis of scRNA-seq data.
Supplementary Table S3;Upregulated GO terms of BP.
Supplementary Table S4;Downregulated GO terms of BP.
Supplementary Table S5;Motif enrichment calculated by FindMotifs tool.

Supplementary Figure S1 Fig. S1 Heatmap of high throughput screening data.

Supplementary Figure S2 Fig. S2 The gene-silencing efficiency and its effect on cell proliferation.

Supplementary Figure S3 Fig. S3 Mivebresib (ABBV-075) inhibited cell viability of CCS cells and lowered expression of EWSR1::ATF1.

Supplementary Figure S4 Fig. S4 The SOX10 knockdown and overexpression efficiency.

Supplementary Figure S5 Fig. S5 A combination of vorinostat and mivebresib is effective for CCS
