# Supplementary material for: Targeting the Clear Cell Sarcoma Oncogenic Driver Fusion Gene EWSR1::ATF1 by HDAC Inhibition
Source: Cancer Res Commun. 2023 Jul 3;3(7):1152–65. doi: 10.1158/2767-9764.CRC-22-0518 (PMC10317042; doi:10.1158/2767-9764.CRC-22-0518)
Supplement: Supplementary Figure S1 — Fig. S1 Heatmap of high throughput screening data. [file crc-22-0518-s02.pdf]

Figure S1.

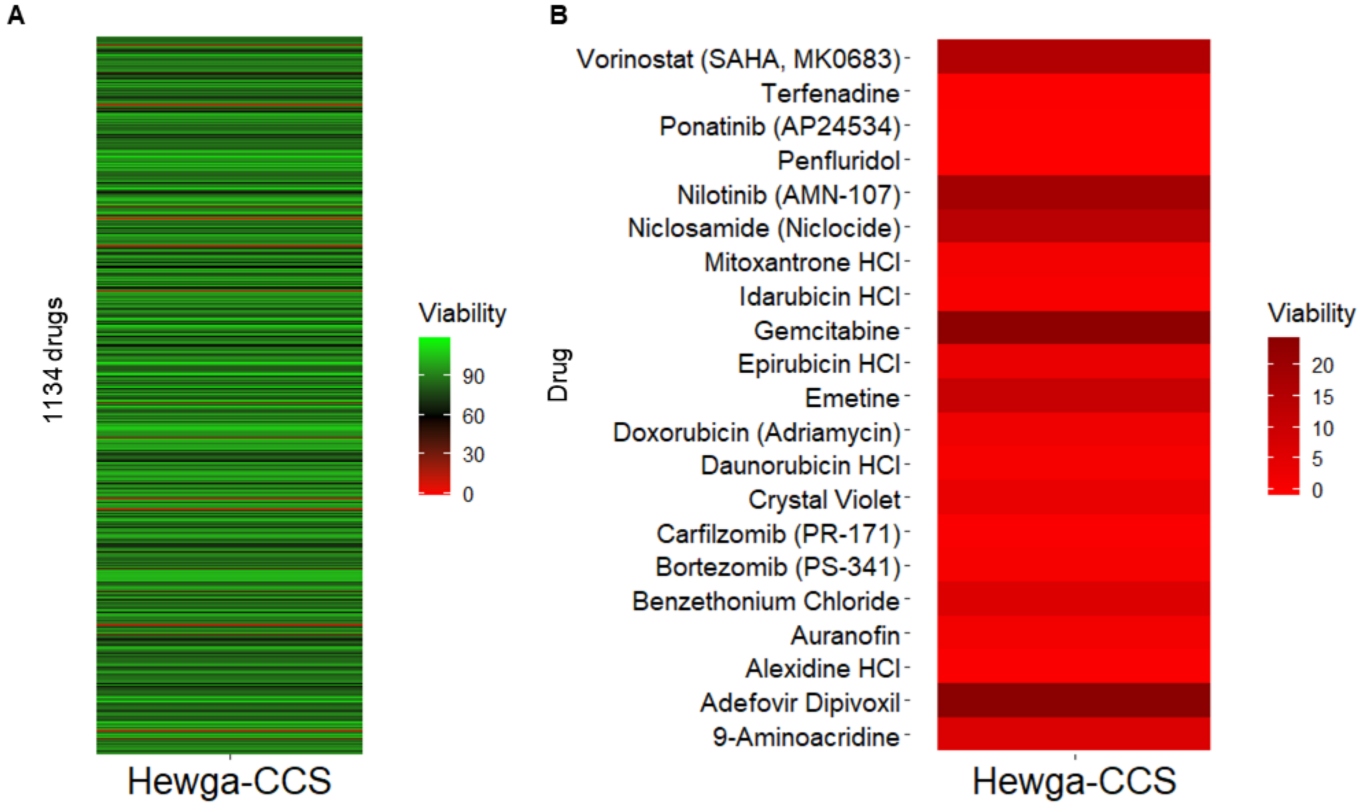

Fig. S1

Heatmap of high throughput screening data.

A, Hewga-CCS cells were treated with 1134 FDA-approved drugs (10 μM each), and the viability of these cells are shown in the heat map. **B**, Drugs that led to a cell viability of <30% are shown in the heat map.
