# Supplementary material for: Targeting the Clear Cell Sarcoma Oncogenic Driver Fusion Gene EWSR1::ATF1 by HDAC Inhibition
Source: Cancer Res Commun. 2023 Jul 3;3(7):1152–65. doi: 10.1158/2767-9764.CRC-22-0518 (PMC10317042; doi:10.1158/2767-9764.CRC-22-0518)
Supplement: Supplementary Figure S2 — Fig. S2 The gene-silencing efficiency and its effect on cell proliferation. [file crc-22-0518-s03.pdf]

Figure S2.

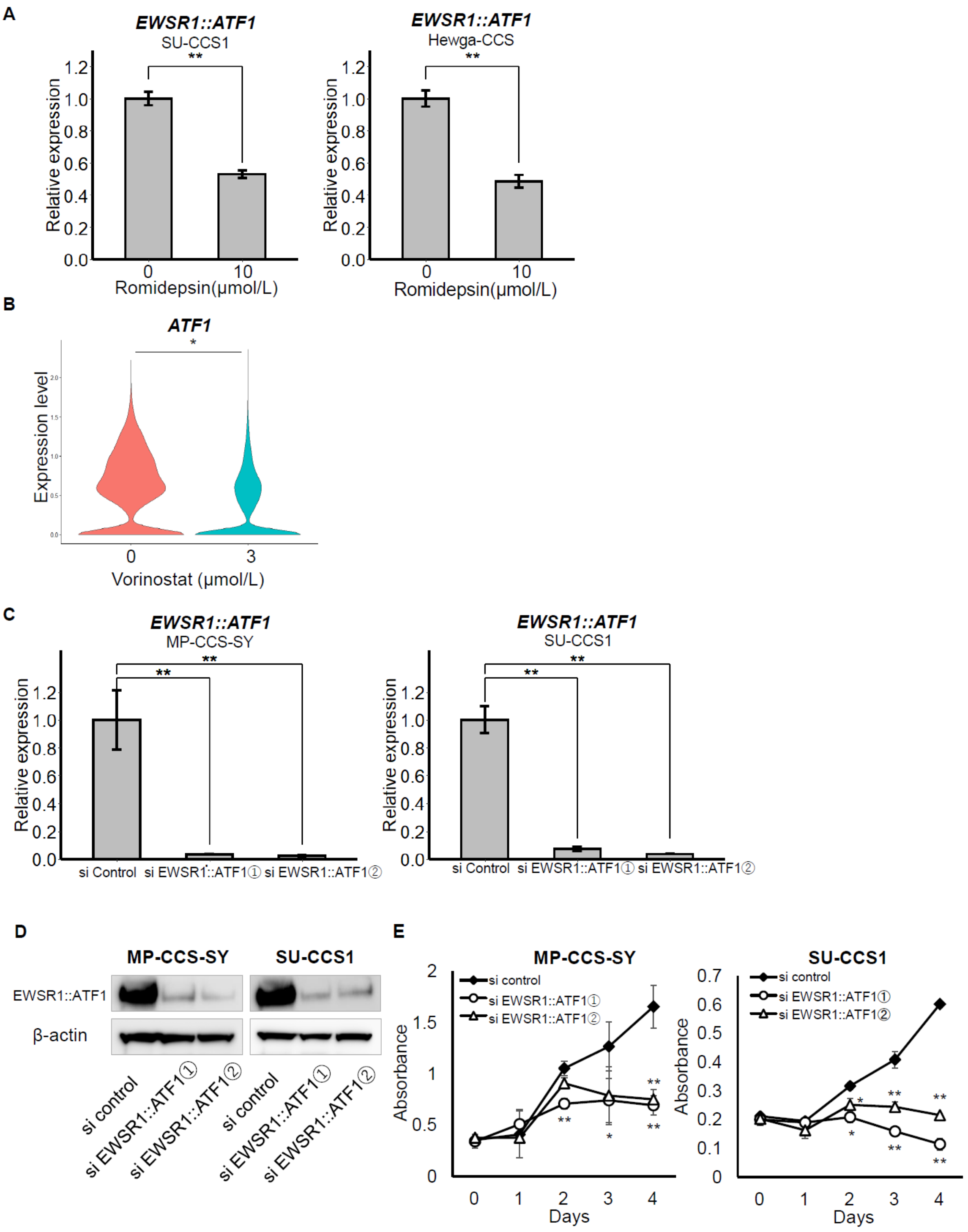

Fig. S2

The gene-silencing efficiency and its effect on cell proliferation.

A, CCS cells were treated with 0 or 10 nmol/L romidepsin for 24 h. *EWSR1::ATF1* mRNA levels in CCS cells were quantified using qRT-PCR (normalized to GAPDH; n = 3). **B**, MP-CCS-SY cells were treated with 3 μmol/L vorinostat or vehicle for 24 h, and EWSR1::ATF1 RNA expression was analyzed via scRNA-seq. **C**, *EWSR1::ATF1* mRNA expression levels of MP-CCS-SY and SU-CCS1 cells 48 h after EWSR1::ATF1 knockdown were quantified using qRT-PCR (normalized to GAPDH; n = 3). **D**, EWSR1::ATF1 protein expression levels of MP-CCS-SY and SU-CCS1 cells 48 h after EWSR1::ATF1 knockdown were detected using western blotting. **E**, Proliferation of CCS cells after EWSR1::ATF1 knockdown were measured with a WST-8 assay during 1–4 days of culture.

Data in **A**–**C** and **E** are means ± SDs. *P < 0.05 and **P < 0.01 (Student's t test).
