# Supplementary material for: Targeting the Clear Cell Sarcoma Oncogenic Driver Fusion Gene EWSR1::ATF1 by HDAC Inhibition
Source: Cancer Res Commun. 2023 Jul 3;3(7):1152–65. doi: 10.1158/2767-9764.CRC-22-0518 (PMC10317042; doi:10.1158/2767-9764.CRC-22-0518)
Supplement: Supplementary Figure S3 — Fig. S3 Mivebresib (ABBV-075) inhibited cell viability of CCS cells and lowered expression of EWSR1::ATF1. [file crc-22-0518-s04.pdf]

Figure S3.

A

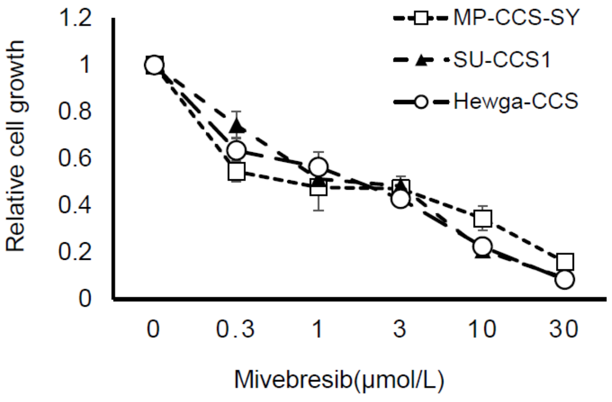

Cell line	IC50s(μmol/L)
MP-CCS-SY	0.67
SU-CCS1	1.67
Hewga-CCS	1.68

B

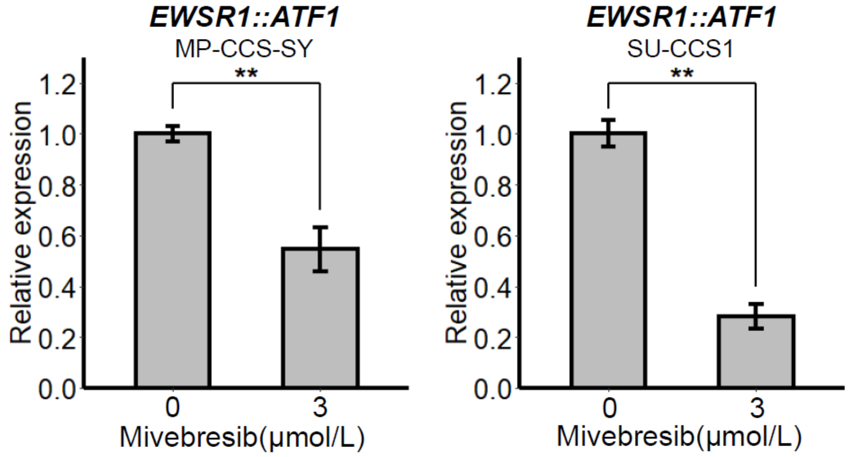

Fig. S3

Mivebresib (ABBV-075) inhibited cell viability of CCS cells and lowered expression of *EWSR1::ATF1*.

A, MP-CCS-SY, SU-CCS1 and Hewga-CCS cells were incubated with 0–30 μmol/L mivebresib for 48 h, and cell viability was estimated via WST-8 assay (n = 3). The calculated IC_{50} values are shown in the table. **B**, CCS cells were treated with 0 or 3 μmol/L mivebresib for 24 h. *EWSR1::ATF1* mRNA levels in CCS cells were quantified using qRT-PCR (normalized to GAPDH; n = 3).

Data are means ± SDs. **P < 0.01 (Student’s t test).
