# Supplementary material for: Targeting the Clear Cell Sarcoma Oncogenic Driver Fusion Gene EWSR1::ATF1 by HDAC Inhibition
Source: Cancer Res Commun. 2023 Jul 3;3(7):1152–65. doi: 10.1158/2767-9764.CRC-22-0518 (PMC10317042; doi:10.1158/2767-9764.CRC-22-0518)
Supplement: Supplementary Figure S4 — Fig. S4 The SOX10 knockdown and overexpression efficiency. [file crc-22-0518-s05.pdf]

Figure S4.

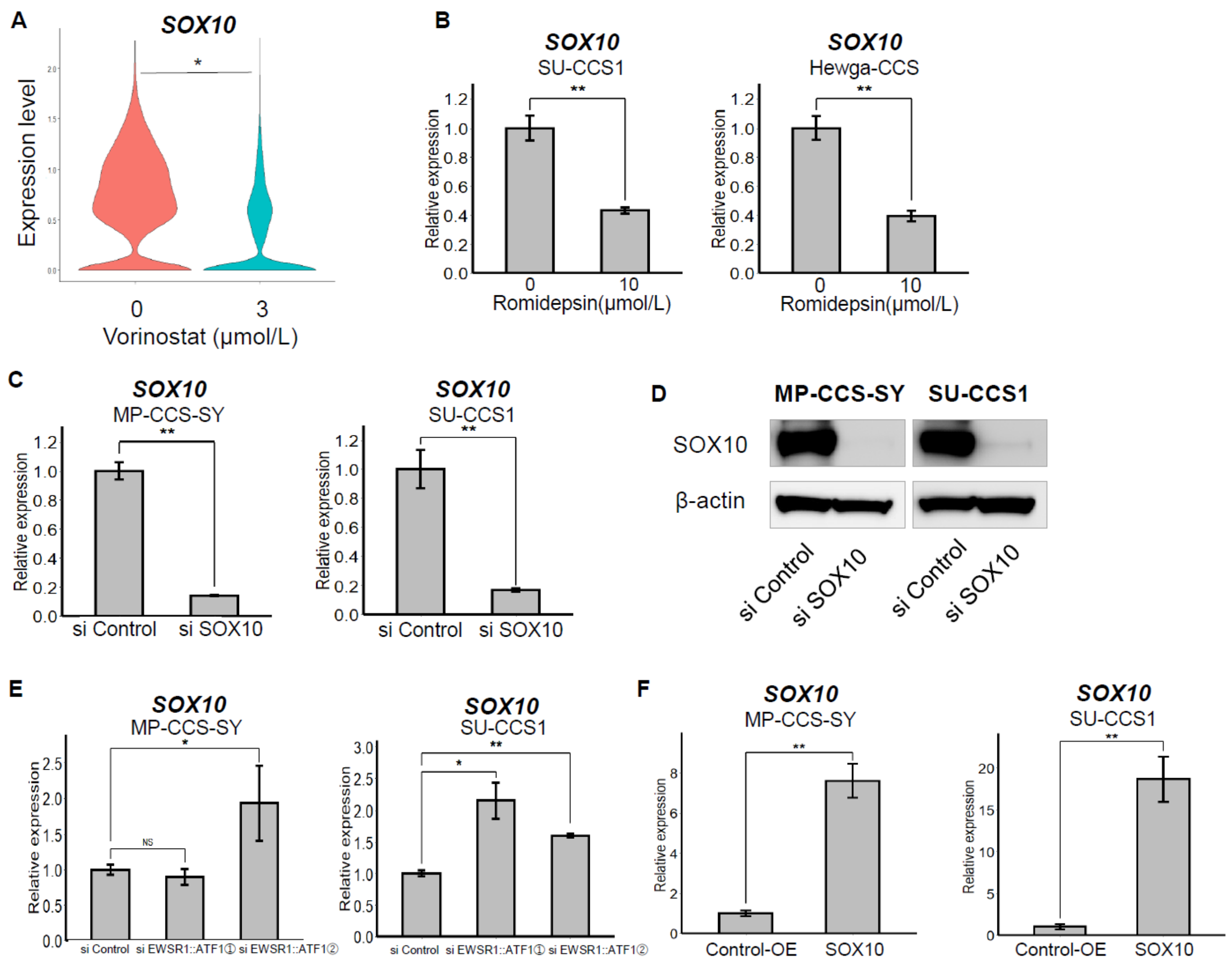

Fig. S4

The SOX10 knockdown and overexpression efficiency.

A, MP-CCS-SY cells were treated with 3 μmol/L vorinostat or vehicle for 24 h, and *SOX10* RNA expression was analyzed using single-cell RNA-seq. **B**, CCS cells were treated with 0 or 10 nmol/L romidepsin for 24 h. *SOX10* mRNA levels in CCS cells were quantified using qRT-PCR (normalized to GAPDH; n = 3). **C**, *SOX10* mRNA expression levels of MP-CCS-SY and SU-CCS1 cells 48 h after SOX10 knockdown were quantified using qRT-PCR (normalized to GAPDH; n = 3). **D**, SOX10 protein expression of MP-CCS-SY and SU-CCS1 cells 48 h after SOX10 knockdown were detected via western blotting. **E**, *SOX10* mRNA expression levels of MP-CCS-SY and SU-CCS1 cells 48 h after EWSR1::ATF1 knockdown were quantified using qRT-PCR (normalized to GAPDH; n = 3). **F**, *SOX10* mRNA expression levels of MP-CCS-SY and SU-CCS1 cells 48 h after SOX10 overexpression were quantified using qRT-PCR (normalized to GAPDH; n = 3).

Data in **B**,**C**,**E** and **F** are presented as means ± SDs. *P < 0.05 and **P < 0.01 (Student's t test).
