# Supplementary material for: Targeting the Clear Cell Sarcoma Oncogenic Driver Fusion Gene EWSR1::ATF1 by HDAC Inhibition
Source: Cancer Res Commun. 2023 Jul 3;3(7):1152–65. doi: 10.1158/2767-9764.CRC-22-0518 (PMC10317042; doi:10.1158/2767-9764.CRC-22-0518)
Supplement: Supplementary Figure S5 — Fig. S5 A combination of vorinostat and mivebresib is effective for CCS [file crc-22-0518-s06.pdf]

Figure S5.

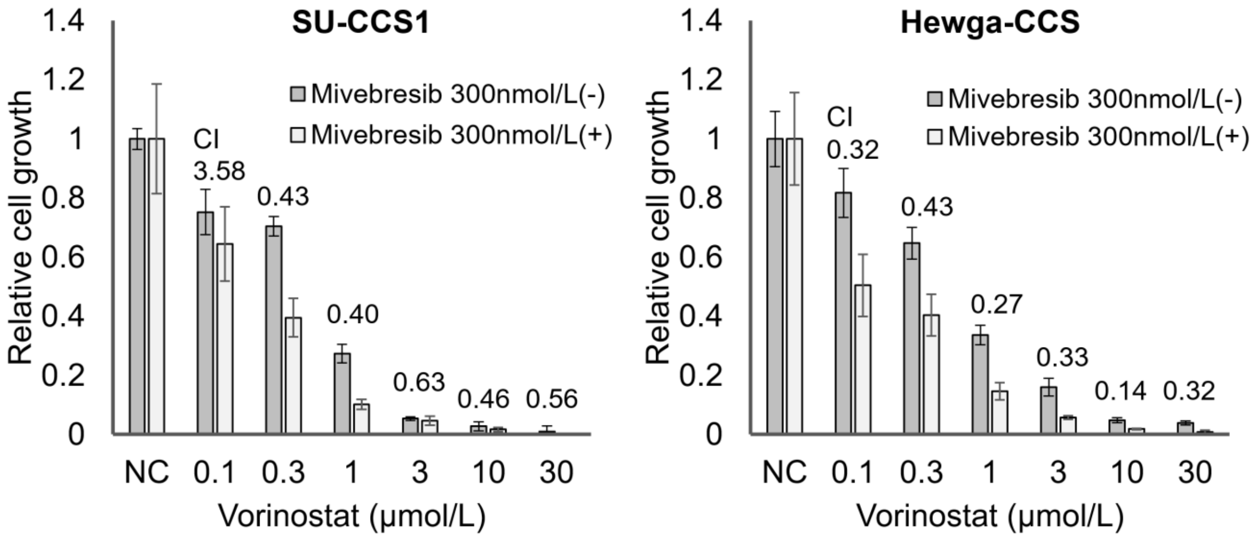

Fig. S5

A combination of vorinostat and mivebresib is effective for CCS

SU-CCS1, and Hewga-CCS cells were treated for 48 h with the indicated concentrations of vorinostat in the absence (–) or presence (+) of 300 nmol/L mivebresib, and cell viability was assessed via WST-8 assay (*n* = 3). The calculated combination index values are shown in the histogram. Data are presented as means ± SDs.
